# Strengthening implementation of integrated care for small and nutritionally at‐risk infants under six months and their mothers: Pre‐trial feasibility study

**DOI:** 10.1111/mcn.13749

**Published:** 2024-10-21

**Authors:** Marie McGrath, Shimelis Girma, Melkamu Berhane, Mubarek Abera, Endashaw Hailu, Hatty Bathorp, Carlos Grijalva‐Eternod, Mirkuzie Woldie, Alemseged Abdissa, Tsinuel Girma, Marko Kerac, Tracey Smythe

**Affiliations:** ^1^ Emergency Nutrition Network Oxford UK; ^2^ Department of Population Health, Faculty of Epidemiology and Population Health London School of Hygiene and Tropical Medicine London UK; ^3^ Department of Paediatrics and Child Health Jimma University Jimma Ethiopia; ^4^ GOAL Ethiopia EW Harargehe Program Area Addis Ababa Ethiopia; ^5^ GOAL Global Dublin Ireland; ^6^ Institute for Global Health University College London London UK; ^7^ Department of Health Policy and Management Jimma University Jimma Ethiopia; ^8^ Armauer Hansen Research Institute Addis Ababa Ethiopia; ^9^ Department of Population Health, International Centre for Evidence in Disability, Faculty of Epidemiology and Population Health London School of Hygiene and Tropical Medicine London UK; ^10^ Division of Physiotherapy, Department of Health and Rehabilitation Sciences Stellenbosch University Cape Town South Africa

**Keywords:** continuity of patient care, Ethiopia, growth, health, infant, malnutrition, mothers, quality of health care

## Abstract

An integrated care pathway to manage small and nutritionally at‐risk infants under 6 months (u6m) and their mothers (MAMI Care Pathway) is consistent with 2023 WHO malnutrition guidelines and is being tested in a randomised controlled trial (RCT) in Ethiopia. To optimise trial implementation, we investigated contextual fit with key local stakeholders. We used scenario‐based interviews with 17 health workers and four district managers to explore perceived feasibility. Eighteen policymakers were also surveyed to explore policy coherence, demand, acceptability, evidence needs, opportunities and risks. The Bowen feasibility framework and an access to health care framework were adapted and applied. Health workers perceived the MAMI Care Pathway as feasible to implement with support to access services and provide care. The approach is acceptable, given consistency with national policies, local protocols and potential to improve routine care quality. Demand for more comprehensive, preventive and person‐centred outpatient care was driven by concerns about unmet, hidden and costly care burden for health services and families. Inpatient care only for severe wasting treatment is inaccessible and unacceptable. Support for routine and expanded components, especially maternal mental health, is needed for successful implementation. Wider contextual factors may affect implementation fidelity and strength. Policymakers cautiously welcomed the approach, which resonates with national commitments, policies and plans but need evidence on how it can work within varied, complex contexts without further system overstretch. A responsive, pragmatic randomised controlled trial will generate the most useful evidence for policymakers. Findings have informed trial preparation and implementation, including a realist evaluation to contextualise outcomes.

## Key messages

- The MAMI Care Pathway integrated approach is perceived as acceptable, needed and possible to implement during a randomised control trial in outpatient clinics in Ethiopia.
- Contextualised inputs (training, staffing, space and supplies) will be needed to strengthen implementation of existing activities and for new components.
- Breastfeeding counselling skills and maternal care provision, especially maternal mental health, are varied and often limited in routine services.
- National policymakers need trial implementation evidence to contextualise RCT outcomes for policy development.

## Graphical abstract

A formative feasibility study investigated factors that may influence implementation of the *MAMI Care Pathway* during a randomised controlled trial in Ethiopia. Health workers perceived it was feasible to implement with support. Policy‐makers need evidence on how it works. Findings strengthened implementation plans and informed a process evaluation to accompany the trial.

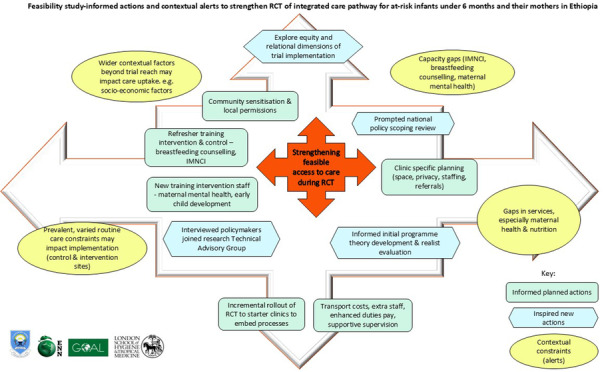

## 1 INTRODUCTION

In low‐ and middle‐income countries (LMICs), an estimated 15.5% of infants under 6 months (u6m) are wasted, 17.4% are underweight, 19.9% are stunted (Kerac et al., 2021) and 14.7% are born low birthweight (LBW) (Okwaraji et al., 2024). Identified and described in many different but closely related ways (McGrath et al., 2024), these infants are at increased risk of poor growth and development, sickness and death with long‐term personal, societal, and intergenerational health consequences (Grey et al., 2021; Lawn et al., 2023; Victora et al., 2008, 2021). Babies may be born nutritionally vulnerable or become so in early life; being born too soon or too small is prevalent and problematic (Lawn et al., 2023) and incidence of stunting and wasting peaks in the first 6 months of life (Victora et al., 2021). Wasting in early life increases the risk of further wasting later (Mertens et al., 2020), contributing to the global burden of 47 million children who are wasted (Global Nutrition Report Stakeholder, 2020).

Ensuring children both survive and thrive is a global health priority reflected in the 2030 Sustainable Development Goals (SDGs) (United Nations, 2015). However, within life‐course interventions to prevent and treat malnutrition (UNICEF, 2021, 2022; UNICEF, FAO, UNHCR, WFP, & WHO, 2021), the care of at‐risk infants u6m has long been overlooked. A 2010 report uncovered a high burden of wasting (Kerac et al., 2011), elevated mortality risk (Grijalva‐Eternod et al., 2017), and widespread policy and service blind spots, particularly for outpatient care (ENN, UCL, & ACF, 2010). The subsequent 2013 update of WHO malnutrition guidelines made transformational recommendations, most notably sanctioning the outpatient management for clinically stable malnourished infants u6m (WHO, 2013). To help put these guidelines into practice, an integrated care pathway approach, the MAMI Care Pathway, was collectively developed in a global expert peer collaboration by the MAMI Global Network (Grey et al., 2023; MAMI Global Network, ENN, & LSHTM, 2021). Modelled on Integrated Management of Childhood Illness (IMCI) guidelines (World Health Organisation, 2014), it applies and expands on what exists to help embed continuity of quality care for small and nutritionally at‐risk mother‐infant pairs within health and nutrition services. Since then, the MAMI Global Network has facilitated implementation research and practical ‘learning by doing’ to help fill priority research gaps (Angood et al., 2015). MAMI Care Pathway principles and practices are reflected in the latest update of WHO recommendations on ‘management of infants u6m at risk of poor growth and development’, a dedicated section of their new management of malnutrition guidelines (World Health Organisation, 2023).

Despite global policy progress, national uptake of WHO recommendations has been low and slow; a 2020 review found that only 6 out of 63 countries recommended outpatient care for infants u6m (Lelijveld et al., 2020). This includes Ethiopia, whose government pioneered and scaled outpatient care for severely malnourished children over 6 months of age (Ferew Lemma et al., 2012) but like other countries, has retained inpatient treatment for infants u6m due to a lack of contextualised evidence on how to do outpatient care (Federal Ministry of Health Ethiopia, 2019). There are also system challenges; while integrated service delivery across nutrition and health is a strategic priority of the Ethiopian government, it has proved elusive partly due to inadequate mainstreaming of nutrition into relevant sectoral policies, strategies, programmes and operational plans (Federal Democratic Republic of Ethiopia, 2021).

To address these critical evidence gaps, the MAMI Care Pathway is undergoing testing in a 4 year (2020–42024) research partnership (MAMI RISE). This involves formative research, a two‐arm, parallel group, randomised controlled trial (RCT) in outpatient health centres in Oromia Region, Ethiopia (https://www.isrctn.com/ISRCTN47300347), realist evaluation and economic evaluation. The RCT will embed a complex public health intervention within existing outpatient health services. Understanding how the MAMI Care Pathway may perform or not in outpatient health facilities is crucial to inform the trial implementation strategy for both the research and intervention components (Pearson et al., 2020). Identifying factors that may impede or enable implementation can improve the conduct and quality of the RCT (Skivington et al., 2021) (Eldridge et al., 2016). Addressing realities in trial implementation also increases the potential to strengthen the relevance and transferability of the MAMI Care Pathway approach to real‐world systems and services (O'Cathain et al., 2013).

The aim of this qualitative study was to improve future MAMI Care Pathway implementation. Objectives towards this were to explore feasibility including acceptability as perceived by health workers and national policymakers in Ethiopia and to contribute evidence on pre‐trial feasibility studies as a means of strengthening contextual fit and improving usability of generated evidence.

## 2 METHODS

We report according to the Standards for Reporting Qualitative Research (SRQR) (O'Brien et al., 2014) and consolidated criteria for reporting qualitative research (COREQ) (Tong et al., 2007). Reflexivity is integrated through methods, results and discussion (Olaghere, 2022). We were consciously aware and declared our ‘vested’ professional roles and the subjectivity this usefully brings to the study. For example, we state the researcher's role(s) in the conceptualisation of the MAMI Care Pathway approach, acknowledge our subjective thought processes and national co‐authors and global practitioners were periodically engaged in the interpretation of data.

### 2.1 Setting

This qualitative study took place in Jimma Zone and in Meta Woreda (district), East Hararghe Zone, Oromia region, Ethiopia. Oromia region has a high burden of child malnutrition; stunting, wasting and underweight prevalence (0–59 months), 35.3%, 4.3% and 16.3%, respectively (Ethiopian Public Health Institute EPHI Ethiopia, & ICF, 2021). The RCT is located in Jimma Zone and Deder Woreda. In Jimma Zone, health workers for this study were selected from centres that would not be included in the RCT to avoid potential bias. Since all health facilities in Deder Woreda are included in the trial, the neighbouring Meta Woreda was selected (similar population and service profile to Deder). Policymakers and key stakeholders were nationally selected from across Ethiopia.

### 2.2 Participants

Health centres staffed by multi‐disciplinary health professionals and health posts operated through health extension workers (HEWs) provide primary care services in Ethiopia (Ministry of Health Ethiopia, 2021).

Purposive sampling identified health workers (nurses and HEWs) for interviews with experience of delivering care to at‐risk infants u6m and mothers in inpatient, outpatient and community health services. We prioritised the staff cadre who would deliver care during the RCT (outpatient clinic nurses). Mothers and families were not interviewed as this was beyond the resource and time available for the formative study; health workers were asked how feasible the approach would be for mothers. Purposive and referral sampling identified policymakers involved in health and nutrition policy development and service planning for survey and/or interview, using researcher contacts and leveraging established relationships.

### 2.3 Data collection

Health worker interviews took place from September to October 2021. Policymaker surveys/interviews took place from September 2021 to March 2022. The RCT commenced 22 August 2022. A UK‐based researcher (BSc, part‐time PhD candidate, Charity Technical Director, female) led the study in collaboration with the MAMI RISE Team. An experienced Jimma University field researcher (BSc, clinical nurse, lecturer Jimma University, and male) conducted the health worker interviews. Both researchers completed training on research ethics and good clinical practice and both training in and experience of qualitative research. The lead researcher administered the online survey and conducted remote interviews with policymakers. In‐country researchers provided administrative and logistical support. Both the lead researcher and field researcher are also members of the MAMI RISE Research Team who are conducting the RCT.

**Figure 1:**
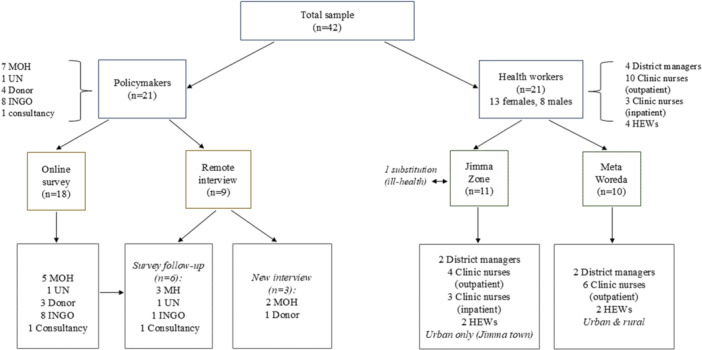
Sampling frame and size. HEW, Health Extension Worker; INGO, International non‐governmental organisation; MOH, Ministry of Health; UN, United Nations agency. District = Woreda.

We interviewed a total of 42 individuals purposively, leveraging local team contacts and connections (Figure 1). We conducted in‐depth interviews with 21 health workers (clinic nurses, HEWs, district (woreda) managers), 11 from urban clinics/hospitals in Jimma town and 10 in Meta Woreda from rural and urban settings. One clinic nurse initially consented to participate but could not (due to sickness). Eighteen senior stakeholders completed the online survey: five Ministry of Health (MOH) representatives, one United Nations (UN) agency, three donors, eight international nongovernmental organisations (NGOs) and one consultancy organisation. Nine interviews were conducted (six had completed the survey and three had not), comprising five MOH personnel, one UN agency, one INGO, one donor and one consultancy organisation. Two UN agency representatives invited for interview were not available (overall 81% response rate).

#### 2.3.1 Health worker interviews

All health workers participated in a 2‐day in‐person orientation on the MAMI Care Pathway and implementation materials within 2 weeks of their interview. The field researcher helped deliver the workshop in both settings. Stakeholder‐specific interview guides were developed in English by the lead researcher in consultation with the field researcher and MAMI RISE Team. Guides were translated into Afan Oromo and Amharic, and locally cross‐checked and piloted with the research team and a clinic nurse (Jimma).

Health worker interviews were conducted by the field researcher in September (Meta Woreda) and October (Jimma Zone) 2021. Having already met interviewees during the workshop, the field researcher re‐introduced himself, his clinical background and his role in this study. Study information was presented verbally and on paper. The field researcher provided information, consented and conducted interviews in Afan Oromo or Amharic as preferred. Interviews were scheduled at the participant's convenience and conducted privately at their place of work. Locally appropriate compensation for 1.5 h of interview time was provided (10 USD mobile data credit). Interviews were recorded and transcribed directly from Amharic or Afan Oromo into English by the field researcher. Transcripts were not returned for interviewee review.

We used a scenario‐based approach in one‐to‐one interviews to explore perceived feasibility. Health workers were invited to apply the *MAMI Care Pathway* materials to two case scenarios (a mother with mastitis who has a 6‐week‐old baby and a mother and her 4‐month‐old undernourished baby) as follows:

1. First, they were invited to use completed assessment forms to classify whether the infant and mother were at‐risk, the nature of risk, decide on actions needed and identify which counselling cards would be used for immediate supportive care. A completed follow‐up form for 1 week hence and at 6 months was then provided and the interviewee was asked to identify which counselling materials would be used at that visit, when to schedule follow‐up and actions at 6 months exit. Health workers were encouraged to share their logic and process as they worked through the scenarios.
2. We also explored how useful they thought the questions would be in helping understand the situation of the mother and her baby, how comfortable they would feel in asking them, and how this compared to their usual practice. All were then asked to score the materials on a scale of 1–5 regarding usefulness, acceptability, practicality and improved care.
3. For district managers, we used the MAMI Care Pathway Framework ‘who, what, where’ schematic to identify where MAMI Care Pathway activities already happen (or not) in the health facilities they manage (Supporting Information: Appendix 1).
4. For both health workers and district managers, we then asked a series of open questions on the appropriateness of applying the MAMI Care Pathway approach in Ethiopia including ‘fit’ with staff roles and responsibilities, perceived acceptability to mothers, what training or support health workers would need for implementation, and what they see as the main implementation challenges in outpatient health facilities. We explored maternal mental health specifically, anticipating challenges informed by earlier formative work and team consultation.
5. Finally, for all we asked who we should talk to about the research to make it work better and invited further ideas and questions.

#### 2.3.2 Senior stakeholder survey and interviews

An online survey (Online Surveys v2, Joint Information Systems Committee) was distributed in English by the lead researcher through email to key contacts and networks across specialties and institutions in Ethiopia (survey live 29 September 2021 to 30 March 2022). The survey asked the extent to which the MAMI Care Pathway approach was needed (demand), its consistency with national health and nutrition policy guidance and plans (consistency), and how possible and how appropriate it was to implement in outpatient settings in Ethiopia including gaps or inconsistencies in the materials and potential opportunities, barriers and harms (acceptability). We also asked what supporting evidence or proof of effectiveness was needed before considering its use in Ethiopia (evidencing policy) and any opportunities in that regard (opportunities). Response options (yes, no, unsure, partially, with adaptation/revision, as applicable) could be further elaborated through free text.

Interviews were conducted as an alternative to the online survey guided by the survey structure (*n* = 3). Survey respondents were also purposively selected for follow‐up interviews where clarification on a response was needed or for deeper exploration on an aspect the lead researcher considered particularly relevant (*n* = 6). All interviewees were encouraged to ask questions and make suggestions regarding the RCT.

Private online interviews were conducted by the lead researcher in English between 25 October 2021 and 22 February 2022. The lead researcher introduced herself, her background (25 years in international nutrition), role in the MAMI RISE Team and lead role in the feasibility study. Interviews were transcribed by the lead researcher aided by automated transcription (otter.ai).

### 2.4 Data analysis

All coding and data analysis was conducted by the lead researcher in the UK. Emerging findings were reviewed with a second researcher (TS) and discussed with the MAMI RISE Team, to inform conclusions and identify implications for the trial.

#### 2.4.1 Health worker data

Health worker interview data were managed and coded using NVivo, v10 software, QSR International, 2012 (K Jackson & Bazeley, 2019). We combined inductive (bottom‐up, theory‐generating) and deductive (top‐down, theory‐testing) approaches to explore observations (Rao & Mandavilli, 2023). Free coding was first conducted to inductively explore the data and identify emerging themes. Deductive thematic analysis was then performed using an adapted version of the Bowen framework (Bowen et al., 2009) to investigate perceived feasibility. This framework unpacks feasibility into eight themes. Relevant phrases were coded guided by illustrative outcomes of interest proposed by the lead researcher to help thematic categorisation (see Table 1). We used the Bowen framework as the primary categorisation and framing for our results. Data were also mapped to an adapted ‘ability to access MAMI Care’ framework (Levesque et al., 2013). This framework takes a person‐centred perspective of both users (mothers) and providers (health care workers) regarding the ability to access care. Aspects informed by applying the ability to access the MAMI Care framework are integrated within and across the Bowen themes. Consequences for RCT planning and implementation are figuratively framed as enabling feasible access to MAMI Care.

**Table 1:** Adapted Bowen framework to investigate the perceived feasibility of MAMI Care Pathway approach.

Area of focus	This study explored	Sample outcomes of interest
Acceptability	The extent to which the intervention is judged to be attractive, appropriate, suitable, satisfying to health workers and to mothers	Consistency with existing policies and services Consistency with planned developments/other initiatives Novelty value Staff workload at health facility Job satisfaction Cultural/social acceptability of the MAMI approach Key decision makers/influencers Possible reasons of how and why it would be acceptable or could be made so *MAMI Care Pathway materials*: format, content, images. nature of questions (content, how they are asked, by whom, cultural acceptability) Rating score
Demand	How much demand is likely to exist for MAMI outpatient care from health workers and mothers	Perceived burden of at‐risk infants u6m Gaps in availability, accessibility in current care Staff desire, (dis)satisfaction Mother/family desire, (dis)satisfaction Consequences of shortfalls in current care Added value to existing services Possible factors affecting demand and differences in demand between different actors in the system and consequences of current shortfalls in care provision *MAMI Care Pathway materials*: Complement/address gaps in existing job aids/guides/resource materials/missing materials
Implementation^a^	The extent to which MAMI Care **could be** successfully delivered by existing staff and services within outpatient health facilities	Stated as 'possible' to implement Consistent with current or planned policies and protocols Consistent with staff roles and responsibilities Existence of necessary health and nutrition services Existence of referral pathways to and from specialist services/community‐based providers Staff/mother's time Implementation strength of routine care Staff competencies and capacity *MAMI Care Pathway materials*: Consistency/added value compared to existing materials
Practicality^a^	The extent to which MAMI Care **would be** successfully delivered using existing means, resources and circumstances and without outside intervention within outpatient health facilities *Identify what may be needed to help accommodate the intervention during RCT*	Functionality of referral pathways Implementation strength of routine care Gaps in service provision External inputs needed for service, e.g. training, equipment, staff External inputs needed for mothers/families, e.g. expenses, time Day‐to‐day operational factors Wider contextual issues *MAMI Care Pathway materials*: Corrections/adaptations identified

^a^ Expansion and integration are inherent in the MAMI Care Pathway RCT, that is, testing an approach being expanded to a new setting (outpatient care) and to a new population (beyond severely wasted infants) integrated within outpatient health facilities.

We applied four of the eight themes of the Bowen framework (acceptability, demand, implementation and practicality). We did not categorise'adaptation’ (modifications), ‘integration’ (system changes) or ‘expansion’ (to different populations/settings) as distinct Bowen themes, since we found these were embedded within acceptability, demand, implementation and practicality themes according to how they were expressed in interviews. These themes are inherent to the MAMI Care Pathway approach (an integrated care model) that is being trialled within existing services in Ethiopia that involves ‘adaptations’ in an ‘expanded’ setting (inpatient to outpatient) and to an ‘expanded’ population (from severely wasted to a mix of vulnerable mother‐infant pairs). These themes will be examined in the RCT realist evaluation. ‘Limited‐efficacy testing’ was not applicable to this type of feasibility study. Implementation strength was considered as what it would take for successful implementation (Hargreaves et al., 2016).

Summary statistics (percentage) using MS Excel were calculated to compare the rating score given by health workers to the MAMI Care Pathway materials.

#### 2.4.2 Senior stakeholder data

Senior stakeholder survey and interview data were analysed and coded to demand, consistency, acceptability, evidencing policy and opportunities, using MS Excel. A tally of the proportion and number of responses by question was made. Narrative synthesis of qualitative survey data was illustrated by anonymised quotes.

### 2.5 Ethical clearance

The study secured ethical approval of the Jimma University Institutional Review Board (IRB) and LSHTM Research Ethics Committee. Permissions from local authorities to conduct the study were secured by the local team. For health workers, informed written consent was secured by the field researcher immediately before each interview. For the survey, online informed consent was mandatory to proceed. Consent to approach for follow‐up interview was sought as part of the online survey and confirmation sought by email in advance of the interview or confirmed verbally at the beginning of the interview, along with consent to record.

## 3 RESULTS

### 3.1 Characteristics of population

Among health workers, professional experience and training varied. Eight reported BSc qualification and one MSc (manager). Over half (*n* = 12) had a minimum of 6 years' practical experience, of whom 6 had more than 10 years. Ten clinic nurses/HEWs had received training on Integrated Management of Newborn and Childhood Illness (IMNCI), breastfeeding counselling, acute malnutrition management and infant and young child feeding (IYCF) to varying degrees; seven reported no further training since primary qualification. Most had not received recent training. All health workers performed well with the scenarios, correctly classifying and accessing support materials during the interview. Professional qualifications and experience were not assessed for policymakers.

### 3.2 Perceived feasibility

**Figure 2:**
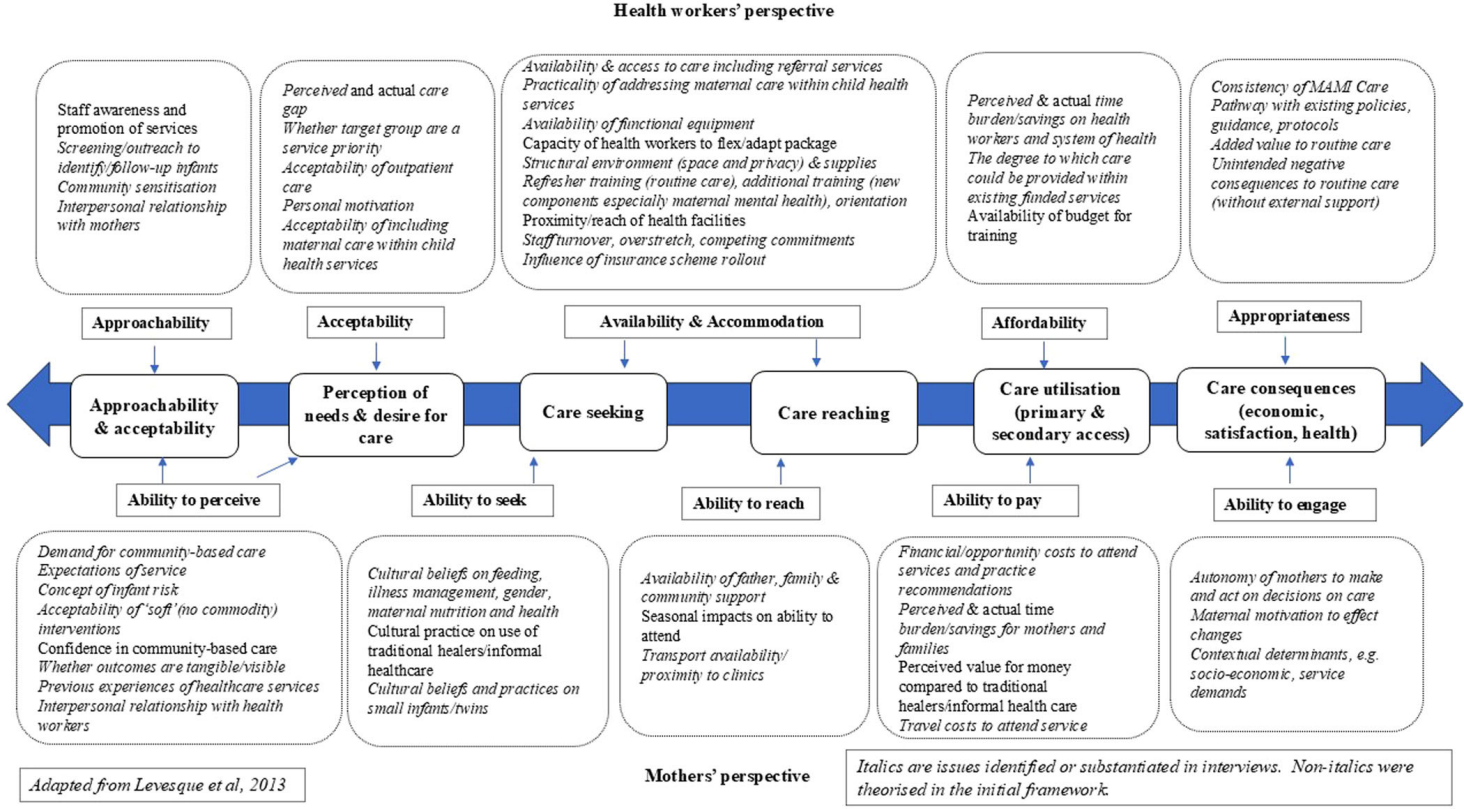
Ability to access MAMI Care framework (updated).

**Figure 3:**
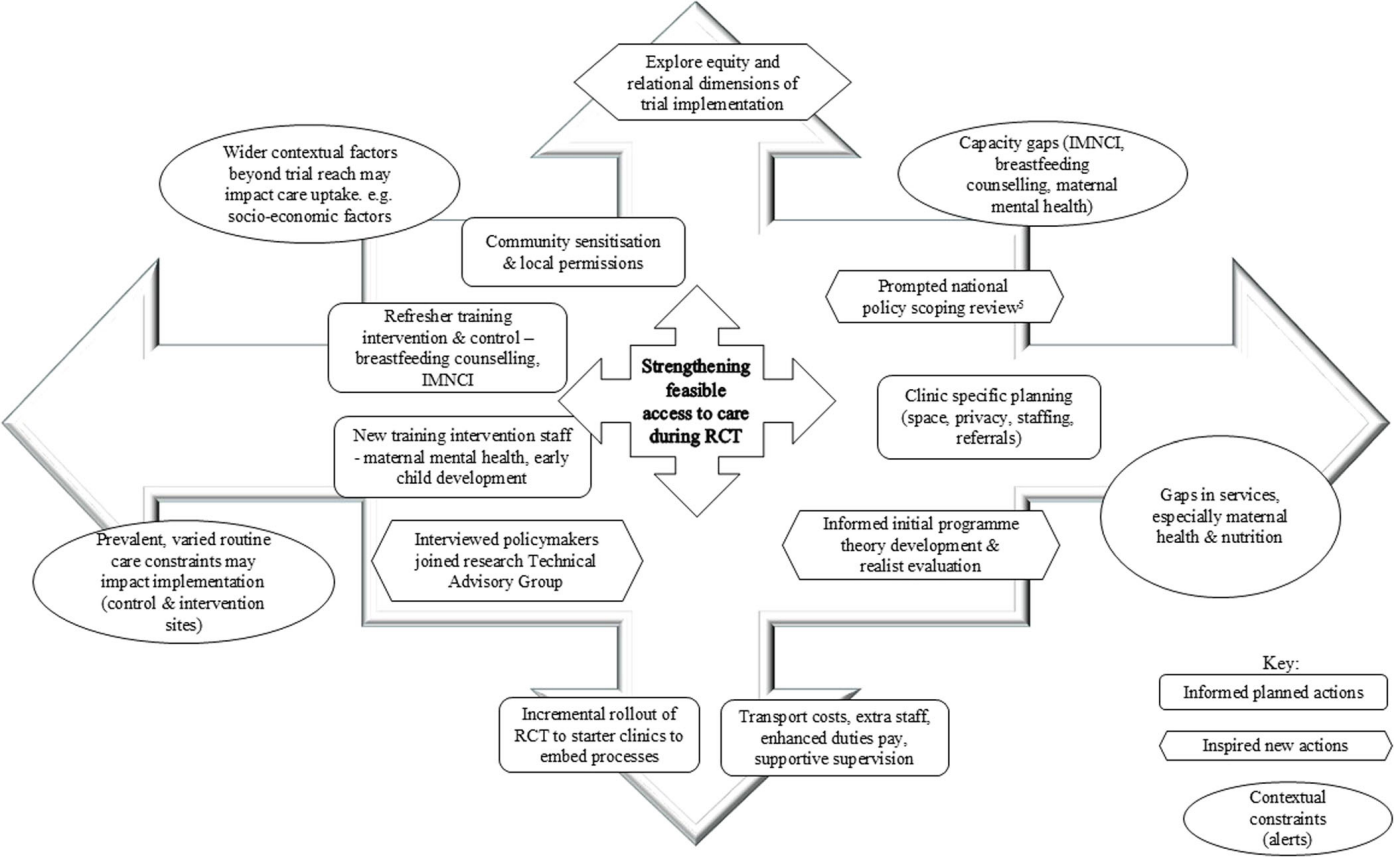
Study‐informed actions and contextual alerts to enable feasible access to care during randomised controlled trials.

Perceptions among health workers and district managers are described by the feasibility theme below. We found considerable interaction and fluidity across and within Bowen themes, which limited strict categorisation by theme or person profile. Summary findings by theme and implications for RCT planning and implementation are provided in Table 2 and Figure 3. Findings were also mapped against the adapted access MAMI care framework (see Figure 2 update).

**Table 2:** Health workers' perceptions of feasibility and implications for RCT preparation and implementation.

Bowen feasibility dimension^a^	Implications for RCT preparation and implementation – research levers and external support^b^
**Acceptability**	
**Health workers** Acceptable as: Offers an accessible alternative to inpatient care for lower risk malnourished infants (inpatient care still needed for high‐risk infants) Help staff deliver more consistent, better, and more efficient routine care Consistent with current policy and practice (IMNCI, iCCM) and potential (to health extension programme) Provides more in‐depth content on breastfeeding counselling Combines prevention and treatment Embeds fundamental maternal care Job aids to address unmet or inconsistently met needs Provides valuable job aids that are culturally acceptable Contributes to professional satisfaction Contributes to a more efficient, less costly system of health **Mothers** Acceptable due to: More accessible (closer to home, reduced transport and opportunity cost) than inpatient care Reduced risks of inpatient care, e.g. increased infection risk, compromised home‐based family care and higher economic burden (direct and opportunity costs). Maternal acceptability may be influenced by: Quality of care (may also influence implementation strength) Lack of medication supplies and equipment that may create dissatisfaction, reduce the uptake of infant services and undermine relationships with health worker Acceptability of fathers and other family members Family planning may be less acceptable to adolescent mothers Cultural influences, e.g. deference to and degree of faith and trust in health workers Degree of understanding of the connection between maternal and infant wellbeing Acceptability of longer and more frequent outpatient appointments would depend on understanding why this care is needed, previous care experiences, evidenced impacts of the intervention (e.g. infant growth) and competing commitments, Not acceptable or may become unacceptable, if opportunity costs are too great, appointments take too much time, perceived benefits reduce as infants recover, Sensitive questions including about her own well‐being may be acceptable to some mothers if they are contextualised to the context of her baby, for others this would not be so. Mothers may be stressed with the condition of her baby or not understand how her wellbeing is linked to her baby's and so not open to probing beyond the baby's condition.	The intervention is likely acceptable to health workers implementing care during the trial and to mothers who are enrolled in the trial **Preparation:** Undertake district and community sensitisation of the trial (why and how) and secure all local permissions **Implementation:** Provide locally approved transport cost for all follow‐up in intervention and for baseline and 6‐month review in control and intervention sites Review and manage waiting times during appointments Engage fathers and other family members in supportive care *Provide information on the ‘why’ of interventions as part of care* Use HEWs to follow up on defaulters *Be alert to any wider issues in health system, such as medication supply stockouts, and repercussions* Encourage feedback/be alert to conversations between mothers on experiences of care during the trial (clinic visits and through realist evaluation) Research ‘levers’ to support implementation in practice should also help improve acceptability (see below).
**Demand**	
**Health workers** There is a strong demand driven by: Perceived burden of care To address needs not met Viable alternative to unacceptable inpatient care Poor job dissatisfaction, guilt due to shortfalls in current care Professional motivation Help more consistently and efficiently do their daily job Hidden cost (time) of trying to sort these infants informally Demand perceived as not shared by health service managers, planners and the community due to: Lack of awareness/recognition of the problem Prioritisation of services for children over 6 months of age Lack of visibility to formal services (no active screening, rely on the presentation of sick babies to clinic, inconsistent/ad hoc growth monitoring, small babies hidden in the community). **Mothers** Less demand is less well recognised as a problem and services not there/obvious to support Demand (and risk recognition) may vary for different infants and mothers, eg infants with disability, dead mother, adolescent mothers, low‐birthweight infants	**Preparation** Sensitisation of clinic managers, district managers, community leaders and health extension workers important to raise awareness and to encourage screening/referral **Implementation** Likely the intervention approach will be well received by health workers who will implement the trial *Be alert to control site staff who may seek input/help if they are dissatisfied with routine care provision or uncertain on how to manage infants identified at risk during trial enrolment* *Study nurses may observe shortcomings in routine care in control sites that may create professional ‘conflict’ on whether to intervene or not*. *Some small infants may not present to clinics*, use health extension worker network to help identify them
**Implementation**	
**Health workers** Consistent with and builds on existing policies and protocols, including iCCM and most notably IMNCI (see Supplementary Table 3). Consistent with outpatient clinic staff roles and responsibilities Intervention may take less time, more time or time neutral that may or may not be acceptable Existing shortfalls in routine care provision such as growth monitoring, due to staff overstretch/multi‐tasking, and referral/communication channels Peak visit days are vaccination days that may create bottlenecks Potential to improve quality of existing care in some clinics, e.g introduce consistency, or compromise it in others, e.g. if staff/system over‐stretched Adolescent mothers may have different needs and cultural considerations, e.g. family planning not so acceptable, less autonomy (raised more frequently by staff from Meta Woreda) Staff experience and confidence to handle sensitive issues, such as maternal mental health, varied. Use of counselling cards not widely used, some use family health cards. **Mothers** Inter‐personal health worker and mother relationship fundamental to quality of implementation Evidence of impact is important for implementation (if mothers do not see improvement, they will not return) but improvement may also dissuade return (if they see improvement, no need to return) Family members and health workers may influence mother's decision and capacity to implement care Implementation intimately linked with acceptability and practicality (see above and below).	**Preparation** *Identify referral pathways on a centre‐by‐centre basis* *Identify space and private area in intervention clinics* *Develop plans for busy vaccination (trial screening) days* **Implementation** *Stepwise rollout of trial in clinics to allow bedding‐in of processes*. *Interpersonal communication skills of study nurses and clinic nurses important in addition to the ‘technical’ know‐how of the activities*. *Prime study nurses to be alert to reports of study activities impacting other services/clinic nurse duties in both intervention and control sites* Provide locally agreed top‐up payment to clinic nurses in intervention sites to reflect extra duties Engage fathers and family members in supportive care *May need to adapt support to specific needs and context of adolescent mothers* Seek feedback from mothers, fathers, health workers and study nurses on experiences of care and research during the trial (research supervision, realist evaluation)
**Practicality**	
**Health workers** Experience and training highly variable, recent training lacking Extensive gaps in staff training and experience to provide mental health support; specialist referral often unavailable Extra support needed: training (IMNCI refresher training, breastfeeding counselling, maternal mental health), additional staff needed, consistent supplies of medications, functional equipment (weighing scales), MAMI Care Pathway materials, therapeutic milk (for referrals) **Mothers** Time, opportunity cost, immediate needs will influence mothers' capacity to implement care Difficult socioeconomic circumstances may greatly impact her capacity to implement care	**Preparation** Refresher training on IMNCI and breastfeeding counselling to control and intervention sites Additional training on MAMI Care Pathway novel components, especially maternal mental health, to intervention sites Provide all MAMI Care Pathway resource materials (translated) **Implementation** *Quality of routine care provision likely to vary across all sites. Staff competencies on breastfeeding counselling likely to vary across sites, that may impact on implementation strength. Some contextualised support may be necessary to address capacity shortfalls in intervention sites to secure implementation strength, to the degree possible within trial resources and scope*. Accommodate extra time for both intervention and for research activities in clinic staff and study nurse duties. Study nurses provide supportive supervision to clinic nurses to address practical challenges. *Explore wider determinants/influences on care during realist evaluation*.
**Feasibility of using the MAMI Care Pathway Package materials**	
The MAMI Care Pathway materials were perceived as feasible to use across all Bowen themes.	
Interviewees appreciated: content (relevance and added components, e.g. adolescent mothers, role of fathers, feeding risk classification) format (checklist, guided management) cultural context (imagery, visuals) added value as a job aid (consistency). Small and easily remedied inconsistencies on terminology relative to existing IMCI‐based protocols were identified: Diarrhoea (MAMI CP) vs. dehydration (IMCI) Cough (MAMI CP) vs. pneumonia (IMCI) Some suggested simplifying the maternal mental health scoring. Scores of perceived usefulness, acceptability, practicality and improved care were high (4/5 for each, 100% response rate (*n* = 21).	

Abbreviations: IMCI, integrated management of childhood illness; IMNCI, integrated management of newborn and childhood illness; RCT, randomised controlled trial.

^a^ Integration and expansion are embedded within acceptability, demand, implementation and practicality according to how they emerged in interviews.

^b^ Research levers/external support were already planned for the RCT. The feasibility study findings supported existing plans (normal text) and identified *nuanced or new considerations (in italics*).

#### 3.2.1 Acceptability

The MAMI Care Pathway approach was perceived as acceptable to health workers, with some exceptions, for many reasons. Most expressed unacceptability of inpatient severe malnutrition treatment (current option), evidenced by poor uptake of inpatient referrals by families. One suggested that given consistency of MAMI Care Pathway with government policy, its ‘*acceptability should not be questioned*’; another felt that policymaker acceptance was a pre‐requisite to acceptability by health workers. General and specific suggestions on key decision‐makers to sensitise ranged from national policymakers to district managers to community leaders.

Health workers perceived mothers' acceptability would be influenced by practicalities and previous experiences of care. Evidence of impact may improve acceptability for some mothers but improved growth may lead to reduced attendance by others. Prevalent medication supply stockouts may negatively impact acceptability by fuelling dissatisfaction, undermining the consultation (*complaining* mothers) and reduce attendance.

Counselling‐based interventions may be less acceptable to mothers experiencing food insecurity or economic hardship, especially if commodity‐based support is common and expected. This was particularly noted by health workers from Meta, which is a more food‐insecure area with a history of external (NGO/UN) food assistance. Suggested ways to improve maternal acceptability (and implementation) of outpatient care included community tracing for defaulters, social support and spending time with mothers (based on experience). Interpersonal relationships between health workers and mothers matter:

If the health professional approaches a mother in a sociable and compassionate way, the mother may share beyond the health issue. My approach is very friendly and due to this, mothers may tell me something private, like a problem she is having in her relationship with her husband. Sometimes I have called the husband and we have talked together as a family to settle some social issue.

Clinic nurse, Meta (MHWR001)

Some mothers may accept being asked about their own wellbeing, appreciating its relevance for their baby's care. For others, this may be unacceptable since too personal, may cause discomfort and mothers may question this scrutiny. Timing of questions may also influence her acceptance:

A mother may be distressed about her child's health problem and asking about her own condition is not timely. It is better to ask these questions during follow up.

Health Extension worker, Meta (MHEW001)

Perceived acceptability of sensitive questions on maternal mental health in the context of infant care varied, influenced by acute and chronic mental health status of mothers, cultural context, and health worker relationship. While a health worker may be comfortable to ask the questions, a mother may not be comfortable to answer them. Many expressed that despite the trickiness of asking mental health questions, they were important to investigate as relevant to infant care and ‘*most of these conditions are not detected unless the mother is asked*’. Some individuals expressed concerns that mental health assessments might lead to over‐diagnosing issues, considering that a mother might be acutely stressed when attending with her unwell baby. Several described how they ‘*put mothers at ease by asking social questions as a gateway*’ to more sensitive issues and in ‘*developing a closer relationship with the mother*’ so that she confides in them.

#### 3.2.2 Demand

Health workers expressed a strong demand for the MAMI Care Pathway approach to address lack of outpatient care in acute malnutrition services for infants u6m and to manage less severe cases. They reported experiencing poor job satisfaction and guilt due to existing gaps. Participants welcomed the proactive combined preventive‐treatment approach; waiting until a baby warrants more complicated medical treatment carries greater expense for families and the health system. The MAMI Care Pathway was needed to help health workers more consistently, comprehensively, and efficiently do their job, to deal with cases some grapple with ‘*daily*’. Most welcomed embedding maternal care with infant care, especially mental health.

Several health workers expressed the need for outpatient care for at‐risk infants u6m may not be shared by health service managers, planners and the wider community. Reasons included lack of awareness and misconceptions regarding the problem, governmental prioritisation of services for children over 6 months of age, and no routine anthropometric screening. Hence, the burden and consequences are ‘*hidden*’ from the formal system that fuels low demand:

Whenever there was a very small infant in the family, the family hid the infant at home and do not visit the health facility. Due to this, there is higher chance of the infant dying because of this condition. MAMI Care Pathway implementation safeguards such small infants.

Health Extension Worker, Meta (MHEW002)

Demand may vary for infants and mothers, influenced by need, recognition of risk and level of risk, e.g. infants with disability, dead mother, adolescent mothers, and LBW infants. Several health workers expressed a strong need to embed maternal care within infant health services for improved, more efficient, accessible and equitable care:

The mother should get services at the same time and contact point as the infant. The MAMI Care Pathway favours integrated care and is appropriate to implement in outpatient care. It is important to help attain equitable health care service delivery.

District manager, Meta (MMAN001)

#### 3.2.3 Implementation

##### Consistency with existing policy and practice

The MAMI Care Pathway was considered highly consistent with existing policy guidance, most citing local IMNCI guidelines that govern outpatient facility care and several referring to iCCM (integrated Community Case Management) that guides community‐level care. In practice, they distinguished how IMNCI focuses on ‘*symptoms and signs of illness*’ and ‘*infant condition and management*’, while the MAMI Care Pathway centres on the mother–infant pair, with ‘*more detailed assessment and support guidance on feeding, maternal mental health and other MAMI risk factors*’ (see further distinctions in Supporting Information: Table 3). Most felt the MAMI Care Pathway approach ‘*fits with staff roles and tasks*’ and that ‘*almost all the activities are what we are performing as routine care*’. The exception was maternal mental health which is not included within IMNCI and ICCM guidance and training packages.

Professional satisfaction emerged as an important personal motivator to implement care. Many described how the MAMI Care Pathway could help improve the quality of routine care, which is often done in an ‘*on‐off way*’ and ‘*overlooked*’, by providing practical job aids to guide assessment and management, resulting in more consistent and comprehensive care:

In routine care we are working without any protocol which is based on each clinician's personal experience. This opens inconsistency among the practice of health professionals. The MAMI Care Pathway helps you not to focus on a single isolated health condition and guides you to think in a multi‐directional way. Otherwise, you may not think of issues related to feeding and your focus may be on signs and symptoms of illness.

Clinic nurse, Jimma (JHWR104)

##### Potential for integration and expansion

Integration with IMNCI was proposed as an ‘*excellent opportunity*’ to embed within routine services that could address ‘*missed opportunities*’ to address mothers and infants together, e.g. screening at vaccination points. Since HEWs are geographically and personally closer to mothers, HEWs were also suggested as having the potential to screen for risk, follow up defaulters, and deliver some components of care. ‘*Awarding educational opportunity, especially for HEWs*’ could be a motivator. Such decentralised ‘*expansion*’ would be consistent with government plans and could enable risk‐differentiated care:

As most of our health centres are going to be scaled‐up to second generation level, managing an infant's condition according to their risk level will be possible in near future.

District manager, Meta (MMAN001)

However, others cautioned staff and services are already overburdened and underdelivering. Another government initiative, expansion of mandatory health insurance currently being piloted, may increase demand for general health services and have a negative knock‐on effect on capacity/supplies for child services.

#### 3.2.4 Practicality

##### Extra time

Perspectives of time costs and consequences for health workers and mothers were intimately linked with acceptability and practicality, with much variation and complexity. Some were already informally spending time on these infants, so introduction was considered time‐neutral:

There is no formal guideline. Health workers are working in non‐formal ways whenever the client has such problems. I don't think that implementation will impact the workload of health professionals.

District manager, Jimma (JMAN002)

For others, extra time taken by health workers would be compensated for by better quality of care, greater job satisfaction and decreased burden on inpatient services by preventing malnutrition, saving time (and costs) to the system. Easing health workers in their daily work may further mitigate time issues, making extra time more acceptable, with implementation becoming more efficient over time:

When IMNCI began, we thought the package would take longer but it was not the case in practice. When the health workers found the package very helpful, no one was thinking about the time it took. Whenever you start something new, there might be challenges which resolve gradually over time.

District manager, Jimma (JMAN001)

For a few, the approach was inconsistent with existing roles due to the extra time needed. Without allocating extra time to staff in already stretched services, care may be compromised, especially for more at‐risk infants:

Most infants who visit our health facilities are those who are seriously ill. They need brief assessment and urgent intervention. Taking longer with one infant may affect the care you give to another.

Clinic nurse, Jimma (JHWR100)

Mothers may need extra time to attend longer and more frequent appointments, which may or may not be possible. Wider contextual factors beyond the health system may influence practicality. Some mothers may accept short‐term time costs given the potential to avoid future greater time/resource costs, for others this may not be possible (nor acceptable) given immediate family and work commitments. Several referred to *Khat* (*Catha edulis, a flowering plant native to eastern Africa with psychoactive properties*) trading that is prevalent in Meta and takes up much maternal time (Cochrane & O'Regan, 2016; Habtamu et al., 2023). Mothers working outside the home may still need social assistance and family support. Several health workers described failed attempts to connect families with the Productive Safety Net Programme (PSNP) (national social protection programme (Abay et al., 2022)) in Meta, with experienced poor child recovery consequences. ‘*Group effort*’ and ‘*commitment*’ was needed for implementation.

##### External support needs

Suggested training/supply needs related to existing shortfalls in routine service implementation, for example, growth monitoring, referral systems, inter‐service information exchange, staffing levels/turnover, and practical constraints, for example, physical space, shortfalls in supplies, and overcrowded vaccination days (see Table 2). Problems may be potentiated if more components are added without support. Compromised care may reflect pragmatic coping strategies:

During vaccination days, health workers just observe the kids physically and if the kid looks emaciated, they link to the health workers working in the under‐five clinic for anthropometric measurement and further assessment.

District manager, Jimma (JMAN002)

Several described insufficient outpatient services for mothers' health, limited to their physical health and ‘*checking for maternal anaemia and screening for malnutrition using Mid Upper Arm Circumference (MUAC)*’. Most identified maternal mental health support would be particularly problematic without extra input, given existing policy and service gaps and may require specialist mental health referrals. Some felt mental health was beyond the remit of outpatient staff dealing with infants, others felt that the staff could be capacitated.

### 3.3 Policymakers' perspectives

A tally of survey responses of senior stakeholders by question is detailed in Supporting Information: Appendix 2 and included with qualitative data from surveys (free text) and interview data below.

#### 3.3.1 Demand

All participants considered the MAMI Care Pathway approach is needed given the ‘*infant and child mortality and malnutrition burden in Ethiopia*’ that necessitates early identification and intervention, ‘*shortcomings*’ in current guidance and services such as lack of accessible outpatient/home care, variable care quality, disconnected services (e.g. between IMAM and growth monitoring), and gaps in job aids for health workers. At‐risk infants u6m and mothers were described as ‘*marginalised*’ within guidance and services; ‘*integrating or mainstreaming*’ the approach within existing guidance was needed to address gaps and to avoid other services being ‘*dropped*’ to make way for something new. Inclusion of maternal mental health was described by one MOH respondent as ‘*very fascinating and promising*’.

#### 3.3.2 Consistency

Policy alignment was full (50%), partial (28%) or unknown (22%). The approach is consistent with government strategy to treat ‘*infants in nutrition need*’. Other consistencies included IMCNI, iCCM, and guidance on mental health, neonatal care, and IYCF. Inconsistencies were not contradictions but reflected additional content in the MAMI Care Pathway, such as maternal mental health, embedding maternal and infant care, and child development.

#### 3.3.3 Acceptability

All stated the approach was needed, 78% that it was possible and that it was appropriate as is (33%) or with revision (56%); ‘*adaption to the complex Ethiopian environment*’ was needed to account for existing services realities. Implementation would require increased staff time, dedicated (integrated) service and space, and training with follow‐up, one noting ‘*every pilot is possible when capacity is supported*’. Several were unsure of its appropriateness given these unknowns.

Half of the respondents identified barriers to outpatient implementation. These are related to human resources (staff numbers, competencies), shortfalls in the quality of routine care, compromised accessibility for some caregivers (social, geographical) and target‐driven health services that may encourage quantity (numbers reached) over quality. Social, service, geographical and cultural contextualisation is needed and expectations of care by users may vary too.

Half felt there were no gaps in the materials, but several cautioned their appraisal was limited by time and expertise. Identified gaps were on performance/evaluation indicators and integration guidance. Several suggested adaptations of existing guidance (IMNCI, iCCM, integrated management of acute malnutrition (IMAM)) to accommodate the MAMI Care Pathway, ‘*harmonising*’ the content that already exists (e.g. feeding assessment) and emphasising the new components, such as mental health (with training). Potential to integrate with iCCM (already at scale through HEWs) would require additional HEW training and/or deployment of extra skilled staff to health posts.

Potential harms (three respondents) were ‘*at system rather than individual*’ level by overstretching staff and service. Identifying workable modifications to address time limitations and first appraising what services (e.g. mental health) are available in different facilities were suggested as mitigating actions.

#### 3.3.4 Evidencing policy

Evidence of in‐country effectiveness was needed by the majority (83%) before implementation, half identified barriers. Investment in implementation research was suggested as ‘*critical*’ to identify barriers to and cost implications of scale. Most wanted evidence on how to implement the approach within different contexts in Ethiopia, citing affordability, adaptability, feasibility, implementation ‘*pitfalls*’ and how to overcome them, service arrangements, acceptability (by users, by MOH). One suggested to evaluate implementation experiences of existing guidelines.

The planned RCT could help identify what contextual adaptations were needed. Evidence generated through the MAMI RISE Project can influence national policy ‘*if MOH are involved in every part of the research process*’, whose endorsement is needed to ‘*activate opportunities for practice*’. Evidence generation could also involve subnational (regional) and cross‐country learning and research.

#### 3.3.5 Opportunities

Most (94%) saw policy/service/practice opportunities to support implementation. National policy, research or practice opportunities ranged from consistency with government goals (to reduce child mortality, hunger, stunting), government aspirations (to identify cost‐effective approaches, comprehensive care provision), and service development plans, such as to upgrade health care provision at selected health posts nationwide. Specific policy opportunities that describe comprehensive care were mentioned (such as the food and nutrition strategy, malnutrition treatment guidelines). Potential to integrate/contribute to updates of guidance on IMNCI, iCCM, maternal and infant and young child feeding, wasting and antenatal care were also identified. Growing attention to early child development and maternal mental health presented an ‘*opportunity to grab the interest of programme managers and policymakers*’.

## 4 DISCUSSION

### 4.1 Feasibility of MAMI Care Pathway will depend on context

We found the MAMI Care Pathway is likely feasible to implement in outpatient health facilities in Ethiopia, if health workers have additional support to deliver quality care and informed families have support to access it. Senior stakeholders' and health workers' perspectives were consistent in terms of perceived need, policy and service alignment and shortfalls in the current system. Most felt the system could accommodate and benefit from the approach but cautioned limitations and risks to care quality of expanding activities without extra support. Support is needed for existing capacity, upon which the approach depends, and for more ‘innovative’ components, particularly maternal mental health. In some ways, the fact that the MAMI Care Pathway is modelled on what exists is a double‐edged sword—it enhances its potential to integrate in existing services, but in practice, is limited by prevailing routine service constraints in Ethiopia (Bayou et al., 2023; Daka, Wordofa & Berhanu, Persson, et al., 2023) and elsewhere, with ‘the policy sacred cow of integrated care repeatedly proving impossible to deliver in practice’ (Greenhalgh & Papoutsi, 2018).

Findings suggest considerable buy‐in for the MAMI Care Pathway approach beyond the context of the RCT. Acceptability of health workers appeared driven by a strong demand to address an unmet need for community‐based, proactive and preventive care that included mothers' wellbeing. This was fuelled by dissatisfaction with ‘hidden’ service and personal costs and the consequences of shortfalls in accessible care. Some touched on organisational readiness in terms of macro and micro motivation and the collective capacity of the system to adopt and sustain the innovation, necessary to facilitate implementation (Domlyn et al., 2021), referring to wider system issues, collective commitment needed and necessary cross‐staff, community and government ‘buy‐in’. Health workers felt that lack of outpatient services for infants under 6 months reflected poor needs awareness and prioritisation by service developers. However, we found that policymakers were needs aware but service development was hampered by lack of evidence on how to expand care provision and to what effect, within already overburdened services.

Consistent with policymakers, several health workers suggested integration of the approach within IMNCI and iCCM training packages. Similarly, both groups identified the potential to expand care provision beyond outpatient clinics, providing some aspects through HEWs that is aligned with government ambitions for more expanded care at selected health post services under the Heath Sector Transformation Plan (HSTP II) (Ministry of Health Ethiopia, 2021). However, given the health extension programme is already ‘at tipping point’, such expansion might help revitalise but would likely need considerable reform to do so (Zerfu et al., 2023; Olaghere, 2022).

Many health workers contextualised their perceptions with ‘if's and but’ qualifiers, reflecting the dynamic and context‐specific nature of feasibility within complex systems for health (Moore et al., 2018) (Marten et al., 2022; Skivington et al., 2021). We found the Bowen Framework a helpful tool to unpack the concept of feasibility and guide a deeper exploration. However, strict thematic classification of data was difficult given the fluidity and interdependence we found between themes and potential feedback loops, including between health workers and mothers. For example, the approach was perceived acceptable to some health workers as it helped address an unmet need (*demand*) and was consistent with and supported implementation of routine care *(implementation*) but would require more staff and training to make it workable (*practicality*). Initial ‘open‐minded’ deductive analysis helped navigate our Bowen‐guided investigation, spotlighting quality of care issues that pervaded Bowen themes; for example, maternal ‘acceptability’ and ‘implementation’ would be affected by mother's previous experience of services, and whether they are treated sensitively, with respect and dignity; their cultural context and the mother‐health worker interpersonal relationship. Using this analytical combination helped to further our understanding on what may shape implementation.

### 4.2 Research co‐creation and partnership strengthens the feasibility potential

Involvement of intended users of implementation research should be considered throughout the research process, from identifying priorities, ensuring implementation strategies are relevant, to disseminating and acting on findings (Harvey et al., 2023; Pérez Jolles et al., 2022). Partnerships between researchers and stakeholders are necessary to achieve sound contextual framing of a new intervention, which requires sustained process investment and multi‐level brokerage (Partnership Brokers Association, 2019). The strong policy and service alignment we found reflects the nature of the MAMI Care Pathway approach that was collectively developed and modelled on IMCI and the strong co‐creative partnership between the MAMI RISE research partners that regularly consult with intended national and international users. From the conceptual stage, planning had a future scale in mind (WHO & ExpandNet, 2011), guided by a scaling framework (MSI, 2020) and secured fair allocation of resources across partners (Lavery & IJsselmuiden, 2018). Our findings demonstrate the value of co‐creation, partnership and early planning for scale to strengthen contextual fit and future scale potential of complex health interventions (Zamboni et al., 2019) and the potency of combining implicit, contextual and global knowledge to help do so.

### 4.3 Implications for RCT preparation and implementation

Among policymakers and health workers, none questioned whether the proposed approach would work. However, all wanted more evidence (or support) on how to deliver it within existing service realities. These findings resonate with and build on team knowledge and formative work to date (Jibat et al., 2022). They endorse the MAMI RISE approach that combines contextual and global knowledge and experience with contextual implementation research to generate more relevant and transformative implementation knowledge (Michaud‐Létourneau et al., 2022), seeking to reduce the evidence‐practice/know‐do gap that prevails in Ethiopia and globally (Daka, Wordofa, & Woldie, 2023; Harvey et al., 2023).

The value of feasibility testing is recognised (Skivington et al., 2021). However, only a quarter of feasibility studies are conducted pre‐trial (O'Cathain et al., 2013). Our study evidences the value of investigating implementation context in preparation for an RCT to help connect the science and practice (Harvey et al., 2023) and access critical local knowledge (Abimbola, 2021). Figuring out how to navigate the system during the trial has greatly benefited from a reality‐check with those working within it (Reed et al., 2018). Here we share what we learned and how it has informed planning and implementation.

#### 4.3.1 Embracing complexity

To generate evidence that improves practice, we need to ‘act scientifically and pragmatically’ while ‘embracing the complexity’ of the setting in which change takes place and ‘engaging and empowering’ those responsible for and affected by the change (Reed et al., 2018). In pursuit of this elusive ‘how’, reacting to rather than just reporting on such contextual realities within the trial will be necessary to generate the most practical evidence and insight that policymakers need (May et al., 2018). Medical Research Council (MRC) guidance encourages such an approach that varies implementation modality according to context, while maintaining the integrity of the core intervention components (Skivington et al., 2021). Unpacking the nature and influence of research levers we use, as well as individual and systems reactions and responses during implementation, will be important to capture through RCT evaluation, to identify ‘nugget’ mechanisms of action and any ‘re‐wiring’ that happens (Ramani et al., 2022), to contextualise findings, inform transferability and bridge the evidence to practice gap (Harvey et al., 2023).

#### 4.3.2 Addressing differing capacity gaps to the degree possible: Reality check

The RCT will rely heavily on existing staff competencies. Already planned refresher training was based on an assumed competency baseline; our study identified considerable variability and gaps in the training and experience of clinic nurses. The degree to which training will address this is likely to vary and will be important to examine during trial implementation. The maternal mental health component was considered both the greatest ‘innovation’ of the approach and the most challenging to implement. While training was already planned, supportive supervision and mentoring during the trial will likely be needed.

We identified factors not modifiable in the context of the RCT that may impact implementation fidelity (delivered as intended) (Hasson, 2010) and implementation strength (what it takes to deliver it successfully) (Hargreaves et al., 2016). Embedding mothers' health as part of infant‐focused services is new, acceptable and important but several flagged low prioritisation and limited services to deliver this. RCT consequences will depend on demand, the degree to which they are met and the impact this has on infant outcomes. Difficult socioeconomic circumstances may impede maternal uptake of care and impact outcomes. While it is beyond the scope of the RCT to provide social assistance, we will be alert to any factors enabling or hindering equitable access to care, including self‐care. Whether and how the MAMI Care Pathway risk‐differentiated approach may facilitate equitable access to care will be an important investigative component of the realist evaluation.

Our findings suggest there will be variability in structural and operational clinic contexts such as space, supplies, referral systems, staff competencies and capacity. Some are more easily modifiable, e.g. allocating space, others less tangible or predictable, e.g. individual staff competencies that will manifest through implementation. Financial/time costs will be compensated to a degree with locally approved ‘top‐up’ to clinic nurses and provision of transport costs to mothers for trial visits. Study nurses will provide surge support to clinic staff for research activities, and extra staff capacity will be secured where needed.

Our study reinforced that the trial will take time to normalise within research sites. Reflecting this, the RCT developed a 3 month ‘bedding‐in period’ with stepped rollout to clinics, first in Jimma and then in Deder. This enabled more intensive support and ‘learning by doing’ in the initial phase, to inform subsequent clinic rollout.

#### 4.3.3 Person‐centred care in research in practice

People are at the heart of systems, the ‘intangible software’ that make, shape and break implementation success (Greenhalgh & Papoutsi, 2018; Ramani et al., 2022). Our study emphasised that the micro‐context of the personal relationship between health workers and mothers may be critical and influential to implementation strength and quality (May et al., 2018). Any intervention needs to be sensitive to those human beings at its frontline, to make sure it is a positive experience for them (O'Cathain et al., 2013). This applies to those both receiving care and those delivering it. Our findings emphasise the importance of addressing the inter‐personal aspects of care delivery as part of training and in on‐going support during the trial, especially for more sensitive maternal mental health and for any new staff who will not (yet) have established relationships with mothers. It will be important to watch for and mitigate overstretch and stress of staff that may not only compromise the implementation of the trial and other services but negatively impact staff motivation and wellbeing and can drive of mistreatment of women (Afulani et al., 2020, 2023). A simplified mother–infant‐centred initial programme theory will be explicitly tested in interviews during the RCT evaluation. We will sensitively interview mothers and fathers as the users of care regarding their relational experiences in both control and intervention sites. Person‐centred care also relates to those health care workers delivering care and study nurses supporting the research so we will be alert to their personal experiences and consequences during the research process.

#### 4.3.4 Capturing the hidden costs of not doing

Health workers touched on the ‘invisible’ costs of not formally dealing with at‐risk infants and their mothers in services and the ‘savings’ to families and services if they did; these are invisible, hard to measure and so rarely quantified. During the RCT, evaluation of health workers, mothers and fathers' experiences of care in control sites will be important to uncover the consequences of ‘not doing’ to help contextualise the more observable ‘price of doing’ in the intervention clinics.

### 4.4 Making the case for pre‐trial feasibility studies

Research levers/external support were already planned for the RCT. The feasibility study findings supported existing plans and identified new or nuanced considerations. Exploring perceived feasibility provided us with depth on areas to strengthen in preparation or implementation and has alerted us to factors within and beyond our control that may impact implementation fidelity and strength (see Figure 3). This has informed initial programme theory development for a realist evaluation to accompany the RCT. Interviewing policymakers engendered interest in the trial (O'Cathain et al., 2013); several policymakers subsequently joined the national RCT Technical Advisory Group. It has sparked us to scope national policy in more detail (McGrath et al., 2023), to most strategically and helpfully contribute to national policy intent and action. We highly recommend feasibility studies precede complex health intervention trials.

### 4.5 Strengths and limitations

*Direct consultation*: We directly tested our assumptions on what was needed to support RCT implementation by consulting with frontline health workers embedded within the health services on implementation realities, and with policy‐makers who evidence and develop national policy.

*Embedded researchers*: The study researchers were embedded in the MAMI RISE Project which allowed for efficiencies in the process, such as logistics for interviews, collective analysis of emerging findings and timely, practical application to RCT preparation.

*Generalisability:* As qualitative research, this study is hypothesis generating rather than hypothesis testing; we cannot be sure if our respondents are representative of others, even within Ethiopia. Views are based on preconceptions and past experiences rather than actual experience. Health workers interviewed from Jimma Zone were all currently working in urban health facilities that may have influenced their experiences and perceptions. We appreciate the limitation that we did not interview mothers and families directly in our study but will do so in the evaluation that will accompany the RCT.

*Availability:* Some senior policymakers were not available during the study period due to other urgent commitments, which meant some perspectives were missed within the study timeframe.

*Responder bias*: Interviewees may have hesitated to challenge the approach that was already planned for trial, and to share their reservations with researchers who were part of the RCT research team. Group orientation pre‐interview may have also been biased towards a ‘group think’ and positivity. Views were made from a position of safety in there being no personal implications, such as increased workload since those selected would not implement the trial. We did not investigate the perceived feasibility of undertaking the research activities, only the intervention components so perceived time needs are likely underestimates.

## 5 CONCLUSIONS

We found that policymakers and health workers perceive the MAMI Care Pathway as feasible to implement during an RCT in outpatient health facilities in Ethiopia, but support will be needed to address prevalent constraints in routine care, capacity gaps for innovative components and to enable sustained access to quality care. Wider contextual influences beyond the influence of the RCT may influence implementation fidelity and strength. Research co‐creation, partnership, early planning for scale and a pre‐trial feasibility study have strengthened implementation readiness and scale potential. A responsive RCT that investigates what works and how will generate the most useful evidence for national policymakers.

## AUTHOR CONTRIBUTIONS

Marie McGrath, Melkamu Berhane, Mubarek Abera, Endashaw Hailu, Hatty Bathorp, Carlos Grijalva‐Eternod, Mirkuzie Woldie, Alemseged Abdissa, Tsinuel Girma, Marko Kerac and Tracey Smythe conceptualised the study. Marie McGrath and Shimelis Girma performed the research. Marie McGrath, Shimelis Girma and Tracey Smythe analysed the data. Marie McGrath wrote the paper. Marie McGrath, Shimelis Girma, Melkamu Berhane, Mubarek Abera, Endashaw Hailu, Hatty Bathorp, Carlos Grijalva‐Eternod, MW, Alemseged Abdissa, Tsinuel Girma, Marko Kerac, and Tracey Smythe reviewed the paper.

## CONFLICT OF INTEREST STATEMENT

The authors declare no conflict of interest.

## Supporting information

Supporting information.

Supporting information.

Supporting information.

## Data Availability

The data that support the findings of this study are available on request from the corresponding author. The data are not publicly available due to privacy or ethical restrictions.
