# Supplementary material for: Strengthening implementation of integrated care for small and nutritionally at‐risk infants under six months and their mothers: Pre‐trial feasibility study
Source: Matern Child Nutr. 2024 Oct 21;21(1):e13749. doi: 10.1111/mcn.13749 (PMC11650029; doi:10.1111/mcn.13749)
Supplement: Supplementary file 1 — Supporting information. [file MCN-21-e13749-s002.docx]

**Supplementary Table 3: Distinguishing features of IMNCI compared to MAMI Care Pathway as perceived by health workers**

| **IMNCI** | **MAMI Care Pathway Package** |
| --- | --- |
| Focus on illness/condition | Includes wider risk factors that are preventive/more comprehensive |
| Focus on infant only | Focus on mother-infant pair |
| Distinguishes infants under 2m and 2m-6m | Targets 0 to under 6m as one group |
| Anthropometry: weight-for-age | Anthropometry: weight-for-length (consistent with national malnutrition guidelines), weight for age, MUAC |
| Assesses infant feeding but does not categorise feeding risk | Assesses feeding and differentiates feeding risk |
| Includes breastfeeding counselling | Additional/targeted content on breastfeeding counselling for risks identified |
| In practice, relies on sick baby presenting to clinic | Uses active screening to identify and act on problems early |
| Does not include maternal wellbeing | Includes maternal mental health |
