# Supplementary material for: Strengthening implementation of integrated care for small and nutritionally at‐risk infants under six months and their mothers: Pre‐trial feasibility study
Source: Matern Child Nutr. 2024 Oct 21;21(1):e13749. doi: 10.1111/mcn.13749 (PMC11650029; doi:10.1111/mcn.13749)
Supplement: Supplementary file 2 — Supporting information. [file MCN-21-e13749-s003.pdf]

MAMI Care Pathway - who, what, where

Management of small & nutritionally at risk infants under six months & their mothers

SCREENING

WHAT?

Screen and refer for assessment using:

IMCI and MAMI-specific danger signs checks

Infant growth

Infant feeding

Maternal health and wellbeing

WHERE?

WHO?

GUIDES & FORMS

MAMI Rapid Screening Guide

ASSESSMENT

WHAT?

IMCI and MAMI-specific clinical assessment

Infant growth

Infant feeding

Maternal health and wellbeing

WHERE?

WHO?

GUIDES & FORMS

- MAMI Assessment Guide & Form
- MAMI Feeding Assessment Guide & Form
- MAMI Maternal Mental Health Assessment Guide & Form
- MAMI Enrolment & Follow Up Form

SUPPORT AND MANAGEMENT

MAMI INPATIENT CARE

WHAT?

1. Clinical care to achieve clinical stabilisation.
2. Provide treatment for congenital conditions affecting feeding (e.g. tongue tie), feeding support, and maternal mental health support.
3. If child reaches 6 months of age in inpatient care, conduct 6 month of age outcome review from inpatient facility.

WHERE?

WHO?

GUIDES & FORMS

Refer to existing national guidance on inpatient treatment of infants under six months with wasting and clinical complications

MAMI OUTPATIENT CARE

WHAT?

1. Counselling on **core topics** for all enrolled pairs.
2. Tailored counselling and actions to address **specific risk factors and problems** as required:
   - Clinical care
   - Feeding counselling & support
   - Mental Health and Psychosocial
   - Support (MHPSS) for mothers
3. Monitoring of progress of mother infant pairs and adjust follow up frequency as appropriate.

WHO?

- Clinical care:
- Tailored feeding counselling & support:
- MHPSS for mothers:
- Monitoring of progress:

WHERE?

REFERRAL TO OTHER AVAILABLE SUPPORT SERVICES AS NEEDED, E.G.

GUIDES & FORMS

- MAMI Enrolment & Follow Up Form
- MAMI Counselling Cards & Support Actions Booklet

6-MONTH OF AGE OUTCOME REVIEW

WHAT?

Assessment at 6 months of age.

IMCI and MAMI-specific clinical assessment

Infant growth

Infant feeding

Maternal health and wellbeing

Referral to support services for continued care if required.

WHERE?

WHO?

CONTINUUM OF CARE

- Infant malnutrition → wasting treatment
- Feeding problems → IYCF
- Clinical issues → IMCI

GUIDES & FORMS

MAMI Enrolment & Follow Up Form

ACRONYMS **IYCF –** Infant and Young Child Feeding **MHPSS –** Mental Health and Psychosocial Support **IMCI –** Integrated Management of Childhood Illnesses
