# Supplementary material for: Strengthening implementation of integrated care for small and nutritionally at‐risk infants under six months and their mothers: Pre‐trial feasibility study
Source: Matern Child Nutr. 2024 Oct 21;21(1):e13749. doi: 10.1111/mcn.13749 (PMC11650029; doi:10.1111/mcn.13749)
Supplement: Supplementary file 3 — Supporting information. [file MCN-21-e13749-s001.docx]

| **Supplementary Appendix 2: Survey responses of senior stakeholders on implementation potential of the MAMI Care Pathway in Ethiopia (n=18)** | | | | | | | | | | | | |  | |
| --- | --- | --- | --- | --- | --- | --- | --- | --- | --- | --- | --- | --- | --- | --- |
|  | **Yes** | | **No** | | **Unsure** | | **Partially** | | **With adaption /revision** | | **Total** | | | |
| **Survey question** | n | ***%*** | **n** | ***%*** | **n** | ***%*** | **n** | ***%*** | **n** | ***%*** | **n** | *%* | |  |
| Is the MAMI Care Pathway Package *consistent* with nutrition and health guidelines in Ethiopia? | 9 | *50%* | 0 | *0%* | 4 | *22%* | 5 | *28%* | *n/a* | *n/a* | 18 | *100%* | |  |
| Is the MAMI Care Pathway *needed* to support care for at-risk infants in outpatient settings? | 18 | *100%* | 0 | *0%* | 0 | *0%* | 0 | *0%* | *n/a* | *n/a* | 18 | *100%* | |  |
| Is the approach *possible* to implement in outpatient setting in Ethiopia? | 3 | *17%* | 0 | *0%* | 1 | *6%* | *n/a* | *n/a* | 14 | *78%* | 18 | *100%* | |  |
| Is the approach *appropriate* to implement in outpatient setting in Ethiopia? | 6 | *33%* | 0 | *0%* | 2 | *11%* | *n/a* | *n/a* | 10 | *56%* | 18 | *100%* | |  |
| Are there *gaps or inconsistencies* in the MAMI Care Pathway Package materials? | 3 | *17%* | 9 | *50%* | 6 | *33%* | *n/a* | *n/a* | *n/a* | *n/a* | 18 | *100%* | |  |
| Do you need *proof or effectiveness evidence* before using the approach in outpatient settings in Ethiopia? | 15 | *83%* | 3 | *17%* | *-* | *-* | *n/a* | *n/a* | *n/a* | *n/a* | 18 | *100%* | |  |
| Do you see any *harms* associated with its use in Ethiopia? | 1 | *6%* | 15 | *83%* | 2 | *11%* | *n/a* | *n/a* | *n/a* | *n/a* | 18 | *100%* | |  |
| Do you see any *opportunities* in policy, services or practice to implementation in outpatient settings? | 17 | *94%* | 0 | *0%* | 1 | *6%* | *n/a* | *n/a* | *n/a* | *n/a* | 18 | *100%* | |  |
| Do you see any *barriers* in policy, services or practice to implementation in outpatient settings? | 9 | *50%* | 9 | *50%* | 0 | *0%* | *n/a* | *n/a* | *n/a* | *n/a* | 18 | *100%* | |  |

n/a: not applicable
